# Development and validation of the nomogram to predict the risk of hospital drug shortages: A prediction model

**DOI:** 10.1371/journal.pone.0284528

**Published:** 2023-04-14

**Authors:** Jie Dong, Yang Gao, Yi Liu, Xiuling Yang

**Affiliations:** Department of Pharmacy, The Second Hospital of Hebei Medical University, Shijiazhuang, Hebei, P.R. China; University of Brescia: Universita degli Studi di Brescia, ITALY

## Abstract

**Introduction:**

Reasons for drug shortages are multi-factorial, and patients are greatly injured. So we needed to reduce the frequency and risk of drug shortages in hospitals. At present, the risk of drug shortages in medical institutions rarely used prediction models. To this end, we attempted to proactively predict the risk of drug shortages in hospital drug procurement to make further decisions or implement interventions.

**Objectives:**

The aim of this study is to establish a nomogram to show the risk of drug shortages.

**Methods:**

We collated data obtained using the centralized procurement platform of Hebei Province and defined independent and dependent variables to be included in the model. The data were divided into a training set and a validation set according to 7:3. Univariate and multivariate logistic regression were used to determine independent risk factors, and discrimination (using the receiver operating characteristic curve), calibration (Hosmer-Lemeshow test), and decision curve analysis were validated.

**Results:**

As a result, volume-based procurement, therapeutic class, dosage form, distribution firm, take orders, order date, and unit price were regarded as independent risk factors for drug shortages. In the training (AUC = 0.707) and validation (AUC = 0.688) sets, the nomogram exhibited a sufficient level of discrimination.

**Conclusions:**

The model can predict the risk of drug shortages in the hospital drug purchase process. The application of this model will help optimize the management of drug shortages in hospitals.

## Introduction

As for drug shortage, one definition given by the FDA is "a shortage occurring when demands exceed supply at any point in the supply chain, ultimately creating a ’stock-out’ at the point of appropriate service delivery to the patient if the cause of shortage cannot be resolved in a timely manner relative to the clinical needs of the patient" [1]. Drug shortage is a global problem [2]. There are many reasons for drug shortage, and supply and demand are the most common ones. For example, there is a shortage of drugs in various medical institutions [3, 4]. The specific situation is that after we submit the order, the distribution company cannot deliver some drugs to the hospital within the agreed time. There are many reasons for this, such as traffic problems [5, 6], etc. In this way, the potential risk of stock-outs is formed. Once the stock of drugs is exhausted, it will affect the patient. However, regardless of its reasons, drug shortage has a significant impact on patients as long as it occurs, including inadequate nursing, poor drug safety and treatment results, poor substitution effect or increased side effects, increased adverse events, prolonged hospitalization, increased patient pain, and even death [7–13]. The additional cost caused by the shortage of drugs is also huge as the drug procurement and shortage management impose hundreds of millions of dollars and nearly ten million hours of additional management time every year [14–17].

At the macro level, each country must take specific measures to manage drug shortages [18–20], for example, China’s volume-based procurement (VBP). It is a national policy that the state selects varieties from generic drugs that have passed the quality and efficacy consistency evaluation, "packs" the scattered procurement volume of medical institutions nationwide to form a large-scale group purchase effect, and conducts price negotiations with drug manufacturers at the national level. At the micro level, Although we have taken some measures, such as reporting the shortage, we still need an active strategy to optimize drug management, from passively accepting the shortage report to actively predicting drug shortages, including establishing a prediction model [21].

At present, the prediction related to drug stock-outs mainly uses time series analysis to predict the use of a drug in the future [22, 23]. However, this kind of prediction is generally used to predict the market potential of drugs. It focuses on the time factor; that is, the time series analysis method reflects the trend of a variable over time. Therefore, the model based on logistic regression is a more suitable method for predicting drug shortages. After the model is visualized, it can be more intuitively displayed using the nomogram. Although some studies [24] have reported that logistic regression-based models are used to predict the drug shortage risk in the pharmacy sector, some of these variables are difficult to obtain. In addition, these studies were not comprehensive enough to evaluate the model visualize it, or show its clinical utility. This paper aimed to establish and validate a prediction model based on multiple logistic regression. This model is used to predict the risk of hospital drug shortages. We used the nomogram to intuitively display the shortage probability of drugs and, finally, decide whether to adopt intervention measures.

## Materials and methods

### Drug shortages information and study design

This study was performed at The Second Hospital of Hebei Medical University and retrospectively obtained drug procurement data from 1 to 31 March 2022. The data of this study were obtained from the centralized procurement platform of Hebei Province, the weekly epidemic situation broadcast on hospital news, and on-site collection.

Data meeting the following criteria were included in the study: (a) drugs existing in the procurement platform; (b) complete data; and (c) drugs in the hospital drug catalog. The exclusion criteria were: (a) active pharmaceutical ingredients and (b) narcotic and psychotropic drugs.

This study aimed to establish a model to predict the probability of drug shortages. The drug information was divided into a training set and a validation set according to 7:3. The training set (n = 4836) was used to establish the model, and the validation set (n = 2077) was used to test the diagnostic performance of the model. Approval of the ethics committee of the hospital was not required for this study.

### Data collection and operational definition

Information on drug procurement and shortage factors was obtained from the Hebei Province Central Procurement Platform, weekly epidemic broadcasts in the hospital news, and on-site survey at the receiving department. A total of 11 variables were evaluated. These variables included volume-based procurement (yes/no), therapeutic class, drug dosage form, distribution firm, order date, take orders (in time/not in time), unit price, COVID-19 (whether the manufacturer is in the COVID-19 epidemic area), manufacturer (foreign-funded/Chinese funded), controlled drug list (whether the drugs are in the list of hospital controlled drugs), and number of continuous stock-out times. Drug therapeutic classes were divided into antibacterial drugs, drugs of the respiratory system, hormone-electrolyte-vitamin (HEV) drugs, antineoplastic, immunomodulatory drugs, endocrine drugs, drugs of the nervous system, drugs of the digestive system, drugs of the blood system, drugs of the circulatory system, and others. Drug dosage forms were divided into injection, oral, and others. The distribution firms were divided into three echelons according to business volume. The first echelon consisted of Sinopharm holding Hebei Pharmaceutical and Le Ren Tang; the second echelon consisted of China Resources Hebei Medical University, China Resources YiSheng, Hebei LongHai, and Hebei General; and the third echelon was the other distribution companies. The purchasing and ordering time was from Monday to Friday. Taking orders in time was defined as the time of taking orders and the time of submitting orders on the same day. Drug prices entered the regression analyses in the form of quintiles. The information on whether the manufacturer was in the middle- or high-risk area according to the weekly report of the epidemic was collected. The number of continuous stock-outs of drugs was collected at the site where the drugs were stored. According to the data distribution, it was defined as zero time and > = one time. The outcome was defined as follows: it was shortages if the quantity of delivered drugs was lower than the order quantity and not if they were equal.

### Statistical analysis

Categorical variables were expressed as percentages, and continuous variables were presented as medians. Univariate logistic regression analyses were performed in the training set to identify independent risk factors. The variables with *P* < 0.05 in the univariate analyses were analyzed in multivariate logistic regression analyses. The final model selection was performed using a backward step-down selection process with the Akaike information criterion (AIC). After that, a nomogram was constructed using the independent risk factors obtained from the multivariable logistic regression analyses. The receiver operating characteristic (ROC) curve was used to discriminate in the training and validation sets. The calibration curve was used to determine whether the predicted shortage probability of the nomogram was consistent with the actual stock-out probability and to analyze it with the Hosmer-Lemeshow test. The decision curve analysis (DCA) was used to estimate the net benefit and clinical utility of the nomogram. All statistical methods were performed using R version 4.1.2 (http://www.rproject.org) with pROC, calibrate, MASS, rms, foreign, and nricens data packages. *P* < 0.05 was considered statistically significant in all analyses.

## Results

### Drug shortages characteristics

**Fig 1:**
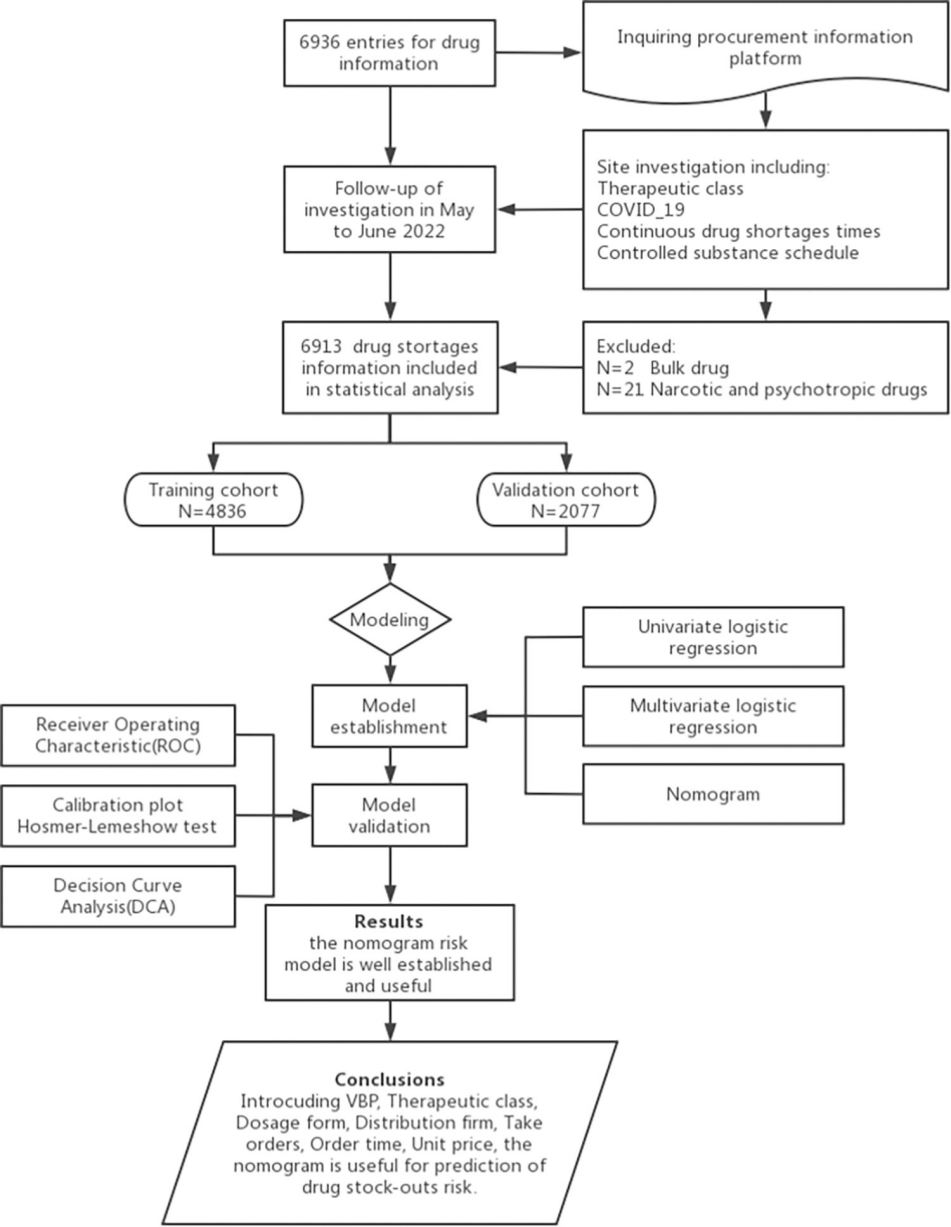
Flow chart of the data selection process.

A total of 6913 pieces of drug information were included in the research, of which 70% (n = 4836) were randomly assigned to the training set, and the remaining 30% (n = 2077) were assigned to the validation set. The selection of drug purchasing information is presented in Fig 1. The drug information characteristics of the training and validation sets are presented in Table 1.

**Table 1:** Characteristics of the training set and validation set.

Characteristics	Training set		Validation set	
	N	%	N	%
VBP				
Yes	520	10.7	227	10.9
No	4316	89.3	1850	89.1
Therapeutic class				
Antibiotics	453	9.4	204	9.8
Respiratory	124	2.6	67	3.2
HEV	644	13.3	270	13.0
Antineoplastic	299	6.2	136	6.5
Immunomodulatory	109	2.3	52	2.5
Endocrine	176	3.6	62	3.0
Nervous system	456	9.4	175	8.4
Digestive system	459	9.5	201	9.7
Blood system	340	7.0	143	6.9
Circulatory system	418	8.6	188	9.1
Other	1358	28.1	579	27.9
COVID-19				
Yes	551	11.4	209	10.1
No	4285	88.6	1868	89.9
Dosage form				
Injection	2287	47.3	998	48.1
Oral agent	2259	46.7	938	45.2
Other	290	6.0	141	6.8
Controlled drug list				
Yes	704	14.6	294	14.2
No	4132	85.4	1783	85.8
Manufacturer				
Foreign	1176	24.3	496	23.9
Chinese	3660	75.7	1581	76.1
Distribution firm				
First echelon	3062	63.3	1332	64.1
Second echelon	1316	27.2	550	26.5
Other	458	9.5	195	9.4
Continuous stock-outs times				
= 0	4599	95.1	1976	95.1
> = 1	237	4.9	101	4.9
Take orders				
In time	3965	82.0	1700	81.8
No in time	871	18.0	377	18.2
Order date				
Monday	1514	31.3	640	30.8
Tuesday	775	16.0	340	16.4
Wednesday	1461	30.2	657	31.6
Thursday	369	7.6	154	7.4
Friday	717	14.8	286	13.8
Unit Price				
Median(range)	52.30(0.39–33180.00)		52.00(0.39–33180.00)	
< = 22.24	949	19.6	438	21.1
22.25–39.80	979	20.2	404	19.5
39.81–71.79	955	19.7	424	20.4
71.80–187.00	990	20.5	392	18.9
> = 187.01	963	19.9	419	20.2

COVID-19, whether the manufacturer is in the covid-19 epidemic area.

### Univariate and multivariate analysis

Univariate analysis showed that volume-based procurement (*P* = 0.000), therapeutic class (*P* = 0.000), dosage form (*P* = 0.000), controlled drug list (*P* = 0.000), manufacturer (*P* = 0.009), distribution firm (*P* = 0.000), take orders (*P* = 0.000), order date (*P* = 0.000), and unit price (*P* = 0.000) were significant risk factors for drug shortages. Related risk factors with *P* < 0.05 in univariate analyses were adjusted for the multivariate analysis. After the multivariate analysis, only volume-based procurement (*P* = 0.000), therapeutic class (*P* = 0.000), dosage form (*P* = 0.000), distribution firm (*P* = 0.000), take orders (*P* = 0.000), order date (*P* = 0.000), and unit price (*P* = 0.000) were still independent risk factors for drug shortages (Table 2).

**Table 2:** Univariate and multivariate analysis of drug shortages in the training set.

Characteristics	Univariate analysis			Multivariate analysis		
	OR	CI	*P*	OR	CI	*P*
VBP	0.49	0.35–0.70	0.000*	0.43	0.29–0.62	0.000*
Therapeutic class						
Antibiotics	Reference			Reference		
Respiratory	0.25	0.12–0.50	0.000*	0.30	0.14–0.66	0.003*
HEV	0.31	0.22–0.43	0.000*	0.25	0.17–0.38	0.000*
Antineoplastic	0.56	0.38–0.82	0.003*	0.98	0.61–1.55	0.919
Immunomodulatory	0.50	0.28–0.90	0.022*	0.60	0.32–1.12	0.108
Endocrine	0.55	0.34–0.87	0.012*	0.91	0.53–1.57	0.733
Nervous system	0.48	0.34–0.68	0.000*	0.54	0.36–0.81	0.003*
Digestive system	0.28	0.19–0.42	0.000*	0.31	0.20–0.49	0.000*
Blood system	0.35	0.23–0.53	0.000*	0.39	0.24–0.64	0.000*
Circulatory system	0.49	0.34–0.70	0.000*	0.67	0.44–1.03	0.068
Other	0.45	0.34–0.58	0.000*	0.43	0.29–0.64	0.000*
COVID-19	1.07	0.82–1.39	0.616			
Dosage form						
Injection	Reference			Reference		
Oral agent	0.76	0.64–0.91	0.003*	0.88	0.69–1.12	0.293
Other	1.22	0.88–1.70	0.241	1.71	1.14–2.55	0.010*
Controlled drug list	1.95	1.58–2.39	0.000*	1.26	0.93–1.71	0.138
Manufacturer	1.32	1.07–1.63	0.009*	1.05	0.82–1.35	0.710
Distribution firm						
First echelon	Reference			Reference		
Second echelon	2.06	1.72–2.48	0.000*	1.66	1.29–2.14	0.000*
Other	2.07	1.58–2.70	0.000*	1.44	1.02–2.01	0.035*
Continuous stock-outs times	4.72e+08	1.62e+71–7.22e+55	0.938			
Take orders	2.68	2.22–3.23	0.000*	1.95	1.49–2.54	0.000*
Order date						
Monday	Reference			Reference		
Tuesday	1.00	0.77–1.31	0.971	0.75	0.56–1.00	0.049*
Wednesday	1.39	1.12–1.72	0.002*	1.28	1.03–1.60	0.028*
Thursday	0.46	0.29–0.73	0.001*	0.43	0.27–0.69	0.000*
Friday	1.24	0.95–1.61	0.110	1.06	0.80–1.41	0.661
Unit price						
< = 22.24	Reference			Reference		
22.25–39.80	0.85	0.65–1.11	0.223	0.68	0.50–0.91	0.011*
39.81–71.79	1.10	0.85–1.42	0.464	0.75	0.56–1.01	0.059
71.80–187.00	1.00	0.77–1.30	0.978	0.64	0.48–0.86	0.003*
> = 187.01	0.71	0.54–0.94	0.017*	0.41	0.30–0.57	0.000*

*Indicates *P* < 0.05; CI, confidence interval; OR, odds ratio; COVID-19, whether the manufacturer is in the COVID-19 epidemic area. Take orders, whether the order is accepted in time (on the same day).

### Construction and validation of the nomogram

**Fig 2:**
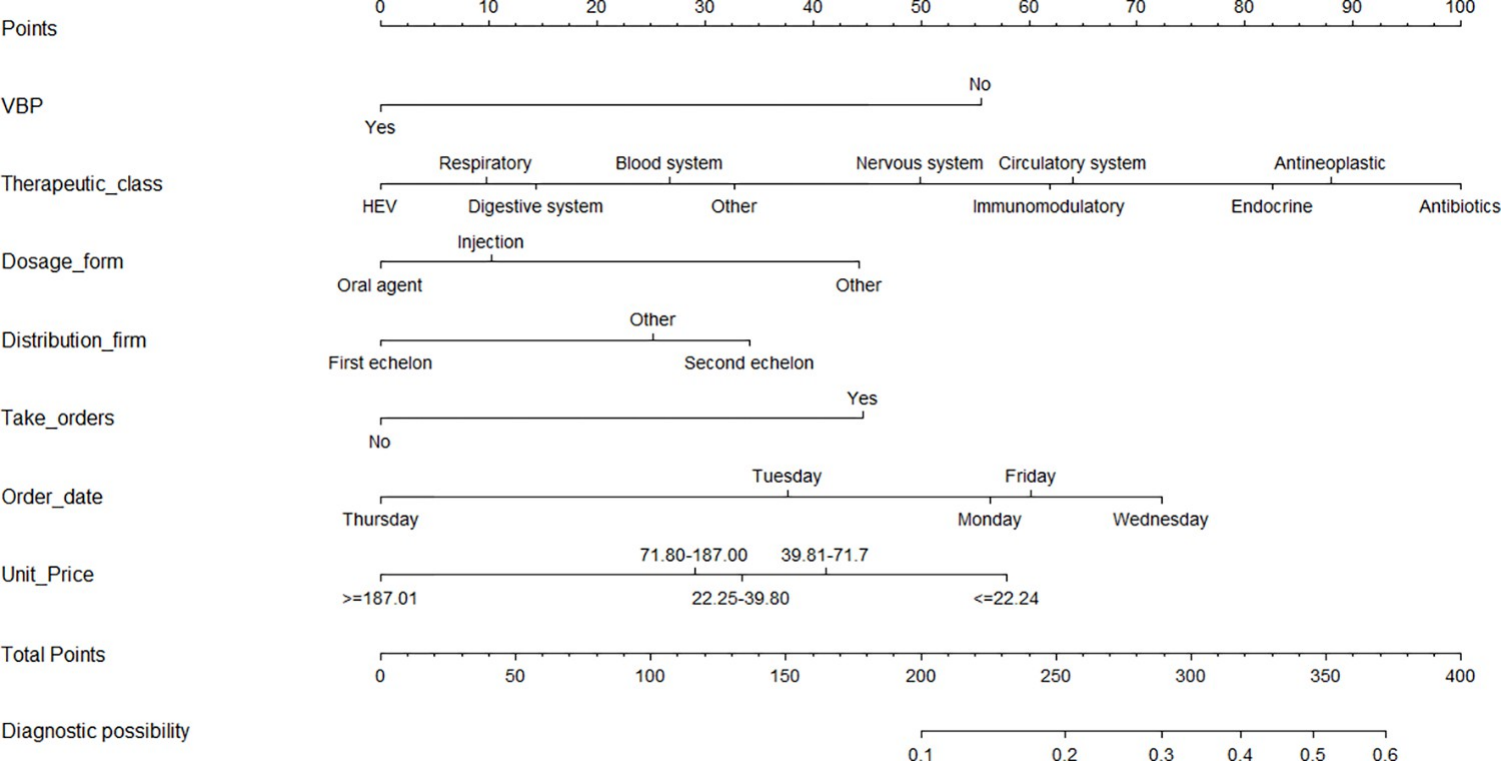
Nomogram for predicting drug shock-outs.

**Fig 3:**
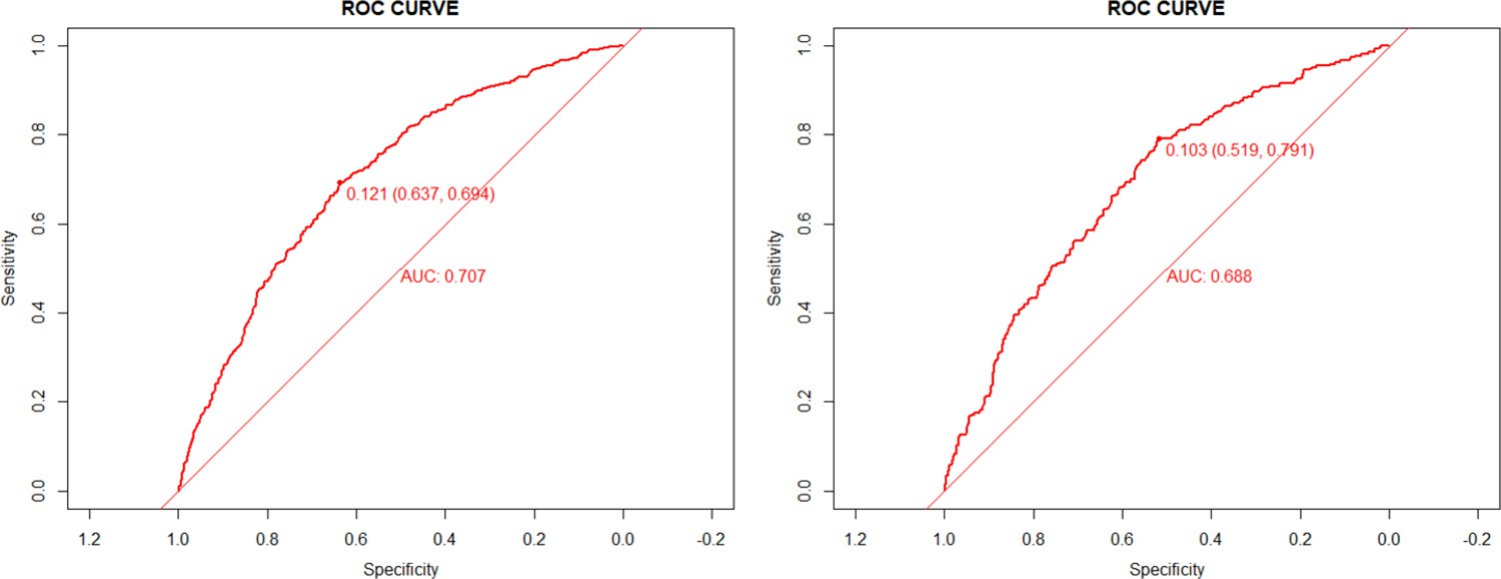
The ROC analyses for the predictive model in training set(A) and validation set(B).

**Fig 4:**
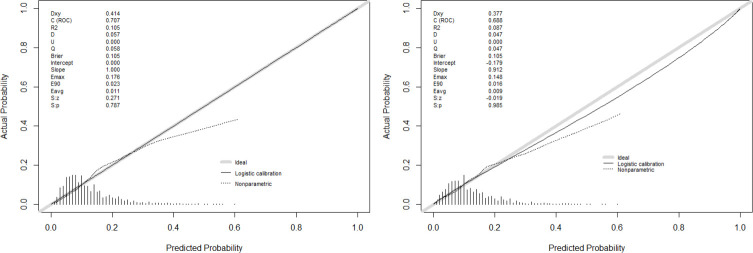
The calibration curve indicates good consistency between training set(A) and validation set(B).

**Fig 5:**
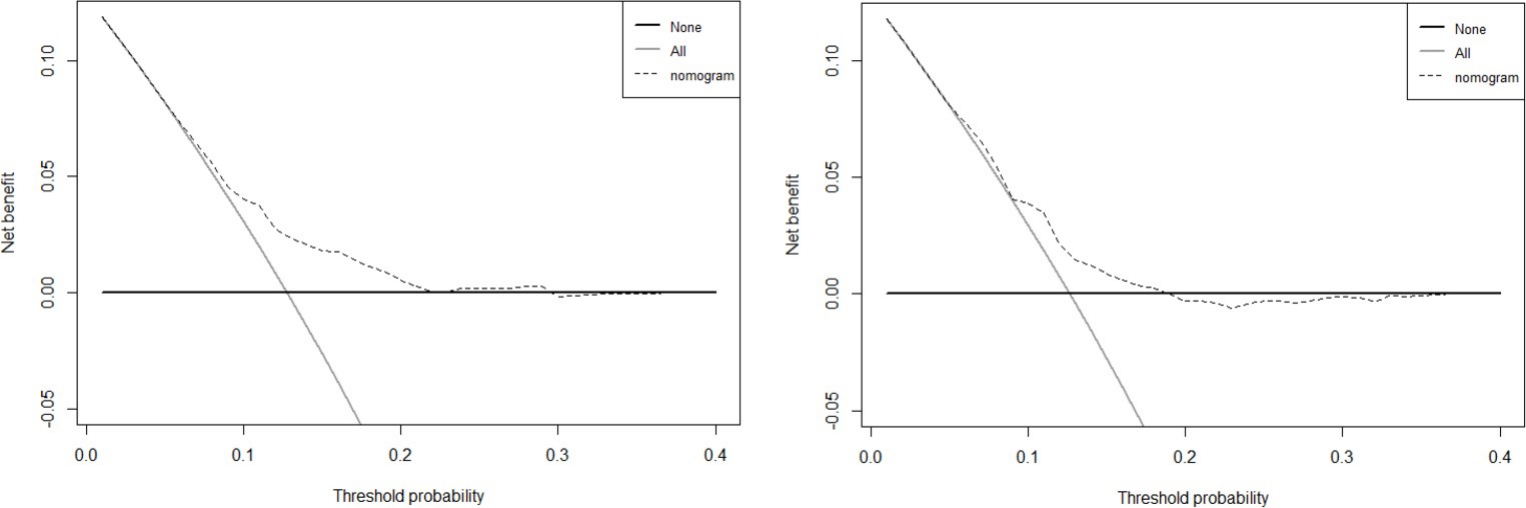
Decision curve analyses for the drug stock-outs nomogram in training set(A) and validation set(B).

According to the results of multivariate analysis, the nomogram for drug stock-outs was established (Fig 2). We identified the score of each independent factor and the total score according to the point scale at the top of the nomogram. Then, we predicted the corresponding stock-out probability according to the total score and the point scale at the bottom of the nomogram. Our analyses showed that the model had a sufficient level of discrimination, with an area under the curve (AUC) of 0.707 (95% CI = 0.686–0.728) in the training set (Fig 3A). AUC in the validation set was 0.688 (95% CI = 0.655–0.722) (Fig 3B). In addition, the calibration curve of the nomogram demonstrated good agreement between the actual and predicted drug stock-out probabilities (Fig 4), and the Hosmer-Lemeshow text showed *P* > 0.05. We used DCA to evaluate the clinical utility and net benefit of the drug stock-out nomogram to further evaluate its potential application worth. These results indicate that more clinical net benefits could be obtained for both training and validation sets (Fig 5). The plot showed that, for predicted probability thresholds between 4 and 29%, model-based decisions had a more net benefit than either the non-interventions or the interventions. The risk factor scores in the nomogram are shown in Table 3. The risk of drug stock-outs was < 10% if the total score was < 200and > 60% if the total score was > 372.

**Table 3:** Scores of prognostic factors in the drug stock-outs nomogram.

Characteristics	Score
VBP	
Yes	56
No	0
Therapeutic class	
Antibiotics	100
Respiratory	10
HEV	0
Antineoplastic	88
Immunomodulatory	62
Endocrine	83
Nervous system	50
Digestive system	14
Blood system	27
Circulatory system	64
Other	33
Dosage form	
Injection	10
Oral agent	0
Other	44
Distribution firm	
First echelon	0
Second echelon	34
Other	25
Take orders	
Timely	0
No in time	45
Order date	
Monday	56
Tuesday	38
Wednesday	72
Thursday	0
Friday	60
Unit Price	
< = 22.24	58
22.25–39.80	33
39.81–71.79	41
71.80–187.00	29
> = 187.01	0

## Discussion

We established and validated a nomogram in this study for predicting the risk of shortages. Our findings showed that the nomogram had favorable discrimination, calibration, and a certain clinical net benefit. Seven predictors were included in the nomogram: volume-based procurement, therapeutic class, drug dosage form, distribution firm, take orders, order date, and unit price. Among them, drug dosage forms and therapeutic classes are consistent with previous reports [24].

Among these predictors, drugs without volume-based procurement were more likely to be shortages than those with volume-based procurement (OR = 0.43). This shows the advantages of volume-based procurement, which can effectively reduce the risk of drug stock-outs [25]. In the drug therapeutic class, antibiotics (reference), antineoplastic (OR = 0.98, *P* = 0.919), immunomodulatory drugs (OR = 0.60, *P* = 0.108), and endocrine drugs (OR = 0.91, *P* = 0.733) were more likely to be shortages than other types of drugs. Among them, antibiotics and antineoplastics were often in short supply worldwide [26–28]. Other studies had shown that endocrine drugs were also among the drugs that were lacking locally [29]. Therefore, we suggest that special attention should be paid to the above four categories of medicines, assessing them before hospital introduction and making contingency plans to deal with stock-outs after hospital introduction. The risk of shortage of other dosage forms (atomized and external drug dosage forms) had a higher risk of shortage (OR = 1.71) than injection and oral agents, which might vary from place to place [30]. Compared with the distribution firms in the first echelon, the distribution firms in the second echelon (OR = 1.66) and others (OR = 1.44) were more prone to the risk of shortage. It might be related to the size of the company and its management strategy. Once it was found in the procurement platform that the distribution firm does not receive orders in time, the risk of shortage will almost double (OR = 1.95). The distribution companies in the first echelon had the lowest risk of shortage, which was consistent with other studies [31]. From this perspective, we should select some large distribution companies (the first echelon) when purchasing drugs, and pay attention to the order information at any time, inquire about the situation, and temporary purchase the drug product from another distribution company if we found that the order was not received on time. In this way, we could minimize the shortage as much as possible. Compared with ordering on Monday, the risk of ordering on Tuesday and Thursday is lower, and the OR was 0.75 and 0.43, respectively. The risk of shortages increased when ordering on Wednesday (OR = 1.28). However, there was no difference in the risk when ordering on Friday (*P* = 0.661). The risk of shortage was the lowest on Tuesday and Thursday, followed by Monday and Friday, and the risk of shortage was the highest on Wednesday. According to the above results, we should try our best to adjust the order time or set up the safety stock to optimize hospital drug management. The unit price of drugs was grouped by quintiles. Compared with the first quintile, the second, fourth, and fifth quintiles were less prone to the risk of shortage, and the OR was 0.68, 0.64,0.41, respectively. In comparison, there was no difference in the third quintile (*P* = 0.059). This means that the higher the price, the less likely it was to be stock-outs [32]. When the drug price is at a low level, it means that the manufacturer should reduce the cost by minimizing the investment [33]. The risk of the drug shortages greatly increases once the price is close to or lower than the cost. Therefore, when drug procurement pays excessive attention to price, the ensuing supply problems should be considered [34].

Some of the factors included in our study were not independent risk factors for drug shortages. The results showed that the COVID-19 factors were not an independent risk factor for drug shortages. The possible reason was that China’s epidemic prevention and control policy was very effective, and China’s strong economic resilience ensured the smooth distribution of drugs [35]. Also, the list of hospital-controlled drugs was not a risk factor for drug shortages. The reason might be that the purpose of formulating the list of hospital-controlled drugs was to prevent the abuse of auxiliary drugs; thus, there was no apparent correlation between drug shortages, even if some distribution firms considered the sales quota of auxiliary drugs and reduced the willingness to deliver. However, the analysis results showed no statistical significance in the risk of shortage of drugs in the controlled drug list compared with drugs outside the list. The study also found no statistical significance in the probability of shortage for both Chinese and foreign-funded drugs. This is inconsistent with some literature reports [24]. The reason may be that the national conditions of each country are different or the volume-based procurement makes it possible to obtain sufficient profit without operation to ensure supply.

In general, using this model may help identify high-risk drugs and the shortage risk per hospital purchase. This can prepare for the next step of planning or implementation of interventions. For example, when updating the hospital drug supply catalog, it can be used to identify high-risk drugs or plan for drug shortages when purchasing. It should also be attempted to reduce the risk of shortage before drugs enter the hospital or undergo the procurement process. In addition, we not only validated the discrimination of the model but also the calibration as well as the decision curve analyses. The model performed well in these validations. Finally, we obtained a nomogram, which could help us easily get a score for a drug as well as the risk of shortage and allows us to quickly evaluate two drugs with different information on the same name. Thus, our model still has limitations. The included independent variables are still not comprehensive. This is mainly because some of these unincorporated independent variables are difficult to collect or quantify, such as the occurrence of the domino effect [36]. Other independent variables cannot be obtained due to the business secrets of the manufacturer, such as raw materials, production line transformation, and whether to expand production. Changes in drug prices can also cause changes in the risk of drug shortage [32]. Therefore, in the future, the consideration of drug price fluctuations and other factors that have not been noticed should be added and included in the prediction model.

As we used retrospective data for modeling, we can use cohort data to continuously test and revise the model in the future. We can also use other medical institutions for external validation to modify the prediction model to achieve better discrimination. In the present study, we used data on hospital drug purchase to establish a prediction model with good prediction ability. This model includes a total of seven variables, all of which are related to hospital drug procurement. It contains some factors reported in the literature and factors not noticed before. After verification, the model performs well in discrimination, calibration, and clinical applicability. Using this model, we can estimate the out-of-stock risk of drugs and decide what intervention measures we need to take. This reflects the initiative of out-of-stock management of hospital drugs. Even in the future, we still need to validate, improve, and expand this model constantly, but the potential of using data modeling for drug shortage management is huge.

## Conclusion

In summary, we have successfully developed and verified a nomogram for predicting the risk of drug shortage based on drug characteristics. The nomogram shows good diagnostic performance, which is very helpful for the hospital to select a drug that is not often out of stock.
